# Proteomic-Based Detection of a Protein Cluster Dysregulated during Cardiovascular Development Identifies Biomarkers of Congenital Heart Defects

**DOI:** 10.1371/journal.pone.0004221

**Published:** 2009-01-19

**Authors:** Anjali K. Nath, Michael Krauthammer, Puyao Li, Eugene Davidov, Lucas C. Butler, Joshua Copel, Mikko Katajamaa, Matej Oresic, Irina Buhimschi, Catalin Buhimschi, Michael Snyder, Joseph A. Madri

**Affiliations:** 1 Department of Molecular, Cellular and Developmental Biology, Yale University, New Haven, Connecticut, United States of America; 2 Department of Pathology, Yale University, New Haven, Connecticut, United States of America; 3 Department of Obstetrics/Gynecology, Yale University, New Haven, Connecticut, United States of America; 4 VTT Technical Research Centre of Finland, Espoo, Finland; University of Giessen Lung Center, Germany

## Abstract

**Background:**

Cardiovascular development is vital for embryonic survival and growth. Early gestation embryo loss or malformation has been linked to yolk sac vasculopathy and congenital heart defects (CHDs). However, the molecular pathways that underlie these structural defects in humans remain largely unknown hindering the development of molecular-based diagnostic tools and novel therapies.

**Methodology/Principal Findings:**

Murine embryos were exposed to high glucose, a condition known to induce cardiovascular defects in both animal models and humans. We further employed a mass spectrometry-based proteomics approach to identify proteins differentially expressed in embryos with defects from those with normal cardiovascular development. The proteins detected by mass spectrometry (WNT16, ST14, Pcsk1, Jumonji, Morca2a, TRPC5, and others) were validated by Western blotting and immunoflorescent staining of the yolk sac and heart. The proteins within the proteomic dataset clustered to adhesion/migration, differentiation, transport, and insulin signaling pathways. A functional role for several proteins (WNT16, ADAM15 and NOGO-A/B) was demonstrated in an *ex vivo* model of heart development. Additionally, a successful application of a cluster of protein biomarkers (WNT16, ST14 and Pcsk1) as a prenatal screen for CHDs was confirmed in a study of human amniotic fluid (AF) samples from women carrying normal fetuses and those with CHDs.

**Conclusions/Significance:**

The novel finding that WNT16, ST14 and Pcsk1 protein levels increase in fetuses with CHDs suggests that these proteins may play a role in the etiology of human CHDs. The information gained through this bed-side to bench translational approach contributes to a more complete understanding of the protein pathways dysregulated during cardiovascular development and provides novel avenues for diagnostic and therapeutic interventions, beneficial to fetuses at risk for CHDs.

## Introduction

The foundations of the primitive heart form between gastrulation and early organogenesis, as critical specification, differentiation, and morphogenic events occur. During this period, the embryo is highly susceptible to environmental insults and genetic lesions that perturb cardiovascular development. These disturbances lead to congenital heart defects (CHDs), a significant cause of spontaneous abortions (20%) and infant mortality (10% of all infant deaths) [1]. Of live births, approximately 1% experience a spectrum of CHDs including transposition of the great arteries, double outlet right ventricle, tetralogy of Fallot, and septal defects [2]. The majority of cardiac malformations (30%) are ventricular septal defects, an abnormal communication between the left and right ventricles [3]. These cardiac anomalies, and several others, arise in part due to morphogenesis defects in the primitive valve tissues (endocardial cushions) which reside in the atrioventricular canal and outflow tract [4]. Within these specific regions of the primitive heart, endothelial cells undergo a key differentiation event, epithelial to mesenchymal transition (EMT) [4]. Lineage tracing experiments and serial anatomical dissection/reconstruction have demonstrated that these mesenchymal cells contribute to the mature mitral and tricuspid valve leaflets as well as the interatrial and interventricular septa [5], [6], [7], [8], [9]. Therefore miscues in the endocardial cushion early in development would affect the ultimate formation of these and other cardiac structures later in development.

Clinical strategies to diagnose cardiac structural anomalies rely on imaging the visible structural defect. Indications for fetal echocardiography include risk factors based on family history, exposures, or other fetal findings [10], [11], [12]. At this point, however, the structural defect has developed and in the current standard of care the clinical intervention is postnatal surgery. Approaching the diagnosis problem from a bed-side-to-bench manner, we believe a molecular-based detection method has the potential to revolutionize the diagnosis and treatment of cardiovascular defects. Therefore a study was designed in which biomarker discovery was performed in a controlled murine model, and then validated for biological significance in human amniotic fluid (AF). A biomarker approach utilizing biochemical analyses of maternal or fetal fluids would provide clinicians with information regarding the pathophysiological condition of the fetus and potentially identify biological deficiencies in the fetal environment. Thus, molecular-based detection provides additional information to guide clinical interventions and may pave the way for the development of novel treatments based on dietary supplementation [13].

The development of such interventions has been hindered by our incomplete knowledge of human cardiac development. A number of genes that drive cardiovascular development have been described in mouse, zebrafish, and chicken model systems, although only a handful of genes such as PTPN11, TBX5 GATA4, and Nkx2.5 have been definitively identified in the etiology of human CHDs [4]. Furthermore, little is known about defects at the protein level. Thus this project aimed to identify novel proteins involved in both cardiovascular development and CHDs.

Identification of proteins involved in specific cardiac developmental events using whole embryonic hearts is difficult because it requires distinguishing and deconvoluting candidate proteins expressed concurrently during endocardial cushion morphogenesis, cardiomyocyte differentiation, looping and/or chamber specification. Therefore, our strategy utilized yolk sac vascular tissue rather than cardiac tissue for proteomic studies. Our hypothesis was that proteins involved in cardiac development and CHDs could be isolated and characterized from the vascular tissue dataset for the following reasons. Yolk sac tissue at the primary capillary plexus stage provides a relatively simple, homogeneous tissue consisting of few cell types in which the primary developmental event is endothelial differentiation and assembly into a mature vascular bed. In the heart, endothelial cell differentiation via EMT is a key event in endocardial cushion morphogenesis. Additionally, trabeculation and coronary system development both also rely on endothelial transdifferentiation or endothelial cell reorganization and assembly. Thus, during development of both the yolk sac and heart, endothelial biology plays a central role. The overlapping developmental pathways in the vasculature and heart have been revealed by the generation of numerous knockout mice that have described both vascular and cardiac phenotypes. This pattern may be a reflection of a common endothelial/cardiac progenitor (though a controversial theory), non-cell autonomous interactions between endothelial and cardiac progenitor lines which are intermingling during migration through the splanchnic mesoderm, or the intimate juxtaposition of differentiated endothelial cells and cardiomyocytes in the embryonic heart [14], [15], [16], [17], [18]. Thus although there is heterogeneity in the type of heart defects that present in the humans there will be a set of markers which will be inherently common to all heart defects because endothelial cell differentiation is a key process in heart development.

From our vascular proteomic dataset, we assembled a list of differentially expressed proteins which were validated by Western blot. The data accrued through these studies highlight the importance of adhesion/migration, differentiation, transport, and insulin/insulin like growth factor (IGF) pathways in cardiovascular development. Immunofluorescence microscopy studies demonstrated the presence of many of these proteins (including Morca2a, TRPC5, Enolase 1, Fbxo42, ST14, and Jumonji) in the embryonic heart. Subsequent functional analysis in an *ex vivo* cardiac culture model revealed novel roles for Collagen IV, NOGO-A/B, WNT16 and ADAM15 in heart development. Together with the immunofluorescence data, the functional analysis data confirmed that our approach did identify proteins relevant to cardiac development. In our dataset, molecules that may be useful as biomarkers in human AF were targeted, in particular secreted proteins and protein ectodomains. The successful application of a cluster of proteins (WNT16, ST14 and Pcsk1) as biomarkers of CHDs was confirmed in a study of human AF samples from women carrying normal fetuses and those with CHDs. This study adds new information to our understanding of the molecular pathways underlying normal and abnormal cardiovascular development, paving the way for the development of novel prenatal screens and treatments for human fetuses with CHDs.

## Materials and Methods

### Mice

CD1 mice (Charles River) were maintained under standard conditions. Timed-matings were detected by the presence of a vaginal plug. NOGO-A/B mice were provided by Steve Strittmatter (Yale University). The Yale University Animal Care and Use Committee approved all animal protocols and experimentation was performed in accordance with National Institute of Health regulations.

### Whole Embryo Culture

Conceptuses were harvested at 7.5 dpc and cultured for 48 hours in pooled rat serum supplemented with 20 µm of the nitric oxide donor NOC-18 (2,2′-(hydroxynitrosohydrazino)bis-ethanamine) (Calbiochem), 10 pg/ml rVEGF (Chemicon), 20 mM L-glucose (control) and/or 20 mM D-glucose. The developmental stage was determined using Downs and Davies' criteria for staging morphological landmarks of the primitive streak, neural plate and head fold [19]. Embryos from head fold stage to somite stage 27–30 correspond to human gestational weeks 3–6 [20], [21], [22]. Embryos were examined under a dissecting microscope for gross defects (neural tube closure, axial rotation completion, yolk sac circulation and heart beat) and processed for mass spectrometry.

### Atrioventricular Canal Explant Assay

Atrioventricular canals were isolated from E9.0 embryos and placed onto a 1 mg/ml Type I Rat Tail collagen gel (BD Biosciences). The explants were allowed to adhere prior to the addition of media [Medium 199, 1% FBS, 100 U/ml penicillin, 100 µg/ml streptomycin, and 0.1% each of insulin, transferrin, and selenium (GIBCO)] [23]. Cultures were stopped at 48–72 hours, fixed with 4% paraformaldehyde and stained with F-actin (Invitrogen) to evaluate cellular morphology. All experiments were repeated at least five times.

### Liquid Chromatography Tandem Mass Spectrometry

Individual 8.5 dpc yolk sacs were lysed in RapiGest SF (Waters), digested with trypsin (Princeton Separations), and desalted using C-18 columns (VivaScience). LC-MS and LC-MS-MS analysis were performed on a Q-TOF API-US quadrupole time of flight tandem mass spectrometer (Waters) equipped alternatively with electrospray and nonospray sources. Peptides were eluted into the source following chromatography on a capillary HPLC CapLC system equipped with a dual wave length UV detector (Waters). For both standard ESI (flow set at 10 uL/min) and nonoflow (flow set at 100 nL/min) ionization methods a linear 30 min gradient of increasing acetonitrile concentration was applied to fractionate and elute the samples. The mass spectrometer's source voltage for this application was set at 3.5 kV with scan time duration of 1 s/scan and spectra were acquired using both survey and parent scan MS methods with data dependent acquisition (DDA) setting defined by MassLynx software instrument interface. Following acquisition, data was preprocessed automatically by MassLynx and the resulting MS-MS spectra was submitted to MZmine for post-acquisition processing (peak picking, data alignment, and normalization) to generate the peak intensity matrix [24], [25]. Data analysis was performed with Matlab and Excel. First the intensity matrix was filtered by dropping away peaks which were detected in less than 5 samples. This left 12908 rows to intensity matrix. T-test (unpaired; two-tailed; equal variances) and log ratio values were calculated for each peak between sample and control independently for all three sample/control pairs: HG vs. control, HG+rVEGF vs. control, and HG+nitric oxide donor vs. control. Log ratios were calculated as average peak intensity in the sample divided by average peak intensity in the control (Table S1). To find peaks with differential intensity between sample and control, threshold levels p-value<0.05 and absolute (log ratio)≥1 were used in each pair. These peaks were targeted (in an aliquot of the original yolk sac lysate sample) for MS-MS fragmentation and their spectra were searched against the nonredundant protein sequence database (NCBI, National Center for Biotechnology) using the Mascot search engine.

### Immunofluorescence

Conceptuses were harvested from timed-pregnant mice at the indicated time points, frozen in OCT medium and cryo-sectioned (10 µm). Immunoreactivity was visualized with Alexa Fluor 594 or 488 secondary antibodies (Invitrogen). Nuclei were stained with DAPI. Digital images were captured on an Olympus IX71 Inverted Microscope.

### Western Blot

Yolk sacs were harvested from culture conditions at the indicated time points and lysed in RIPA Buffer (Upstate) supplemented with 1% SDS, Complete Protease Inhibitor Cocktail (Roche) and Phosphatase Inhibitor Cocktails (Calbiochem). The samples were homogenized and soluble extracts obtained by centrifugation. Protein concentrations were determined by BCA Assay (Bio-Rad) and 25 µg was electrophoresed on SDS-PAGE gels (Bio-Rad). Luminescence was performed using the Western Lightening Chemiluminescence Reagent (PerkinElmer) and densitometry using Quantity One Software (Bio-Rad).

### Antibodies

Primary antibodies included NOGO-A (gift from Steve Strittmatter, Yale), PECAM (J.A.M), Laminin 1 (J.A.M.), α smooth muscle cell actin (Sigma), ADAM15 ectodomain (R&D Systems), WNT16 (BD Pharmigen), SCVT (Slc23a1) (Alpha Diagnostic International), Collagen IV (Abcam and USBiological), PIASγ (Abcam), TRPC5 (Chemicon), KIF21B (Upstate), Erk2 (Santa Cruz), NOGO-A/B (Santa Cruz), Enolase 1 (USBiological), and the following from Novus: Fbxo42, Jumonji, Pcsk1, ST14, CHST3 and ZCWCC1 (Morca2a).

### Zymography

Yolk sacs were lysed in 50 mM Tris/1% Triton-X and homogenized with a motorized pestle. 25 µg of protein was mixed with non-reducing Laemmli buffer and loaded onto a 10% Zymogram Gel (Bio-Rad) for electrophoresis. The gels were washed in 2.5% Triton X-100, followed by water, and incubated for 48–72 h at 37°C in a 50 mM Tris-HCl buffer, pH 8.0, containing 5 mM calcium chloride or 10 mM EDTA (negative control for MMP activity). Gels were stained in 50% methanol, 10% acetic acid containing 0.1% Coomassie Blue R250 and imaged on a BioRad Chemidoc XRS.

### Human Samples

Via ultrasound-guided amniocentesis, 16–22 week gestation amniotic fluids (AFs) were obtained from healthy women (average age 27.7±6.6) carrying fetuses with (n = 17) and without (n = 16) cardiac defects from 2004–2007 at Yale New Haven Hospital. The Human Investigation Committee of Yale University approved our study protocol, and written informed consent for research purpose was obtained from all participants before the procedure. The purpose of the amniocentesis was clinical: to rule out chromosomal abnormalities. Criteria for the “diseased group” were heart defect visualized on at least two ultrasound exams. In cases resulting in live newborns, the defects were confirmed postnatally. In the remaining cases, the pregnancy course was terminated and the defect was confirmed in elected autopsies. CHDs included ventricular septal defects, valvular stenosis, complete atrioventricular septal defects, bilateral ventriculomegaly, hypoplastic ventricle, tetralogy of Fallot, and transposition of the great arteries. Criteria for the “non-diseased group” were AF samples from women undergoing genetic testing who had a normal course of pregnancy including term delivery. All fetuses in the non-diseased group were euploid and had normal heart ultrasound examinations during pregnancy and a normal physical exam at birth.

Frozen AFs were centrifuged and aliquoted prior to assay. Western Blot was performed as described above on 25 µg of AF. A “standard” control sample was chosen to run on each gel. Coomassie staining of gels confirmed equal protein load. Films were scanned and analyzed in Quantity One as follows. Bands were selected for quantification using the automated volume contour tool. Band densities were calculated by computer algorithm as the volume, the sum of pixel intensities multiplied by pixel area, adjusted by global background subtraction. Blotting and analysis was performed by a blinded observer.

Univariate logistic regression was performed using the design packet in R (GNU S). The AF measurements were log-transformed using log2 and the regression coefficient and odds ratio (OR) were calculated. The OR can be interpreted as the increased risk of developing a cardiac defect per doubling of the AF measurement. The mean classification error was calculated using 10 by 10 cross-validation. An attempt to perform multiple regression analysis did result in inflated regression coefficient estimates due to strong multicollinearity. After normalization and centering of all biomarker data, Principle Component Analysis (PCA), a clustering tool that reduces multidimensional data sets to identify new meaningful underlying variables, was performed using the SVD function in Matlab.

## Results

### Tissue Samples from a Murine Model of Induced Cardiovascular Defects

One premise of our study is that proteomic profiling of hyperglycemia exposed vascular tissue will enrich for factors involved in endothelial differentiation or dysfunction. Hyperglycemia was used as an “inducer” because increased vascular and cardiac defects are observed in the offspring of diabetic rodents (streptozotocin-induced) and the infants of diabetic women [26], [27], [28]. Interestingly, pregnant women presenting with altered diurnal gylcemic profiles not related to diabetes also have a higher incidence of preterm labor, fetal malformations and perinatal mortality similar to diabetic patients, underscoring the devastating developmental effects of hyperglycemia [29]. The addition of 20 mM D-glucose (a plasma concentration of glucose often observed in both human and rodent diabetics during a hyperglycemic episode) to *ex vivo* embryo cultures results in vasculopathy in the yolk sac and inhibition of EMT in the cardiac endocardium [23], [30], [31], [32], [33], [34]. These studies contribute to accumulating evidence that transient hyperglycemia directly affects the endothelium, and highlight the importance of endothelial cell homeostasis during embryonic development [35]. Identification of candidate differentially expressed proteins between normal and hyperglycemic groups will provide insight into currently unrecognized proteins in cardiovascular development and may allow for the discovery of biomarkers of CHDs applicable to the general population.

**Figure 1:**
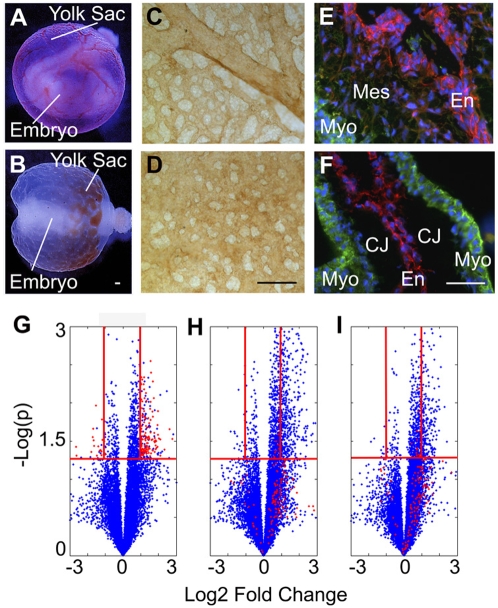
Experimental Model: Applying Proteomic Profiling to a Hyperglycemia Induced Model of Cardiovascular Defects. Embryos cultured *ex vivo* exhibit normal organogenesis and vascular development (A, normal embryo inside a functional yolk sac containing circulating red blood cells). Embryos treated with 20 mM D-glucose display malformation of the embryo and vasculature (B, abnormal embryo and pooling of blood at one pole). PECAM staining of control yolk sacs reveals a mature, remodeled vasculature (C) while hyperglycemic yolk sacs appear immature and mal-developed (D). Normally by E9.5, EMT has begun within the cardiac endocardial cushions (E). Treatment with hyperglycemia results in an acellular endocardial cushion (F). E–F: α-Smooth muscle cell actin (green) labeling of the myocardium (Myo) and mesenchymal cells (Mes); PECAM (red) staining of the endocardium (En); CJ (Cardiac Jelly). Volcano plots (G–I)) depicting each peptide peak detected by LC-MS as a blue dot and plotting the log_2_ ratio (of treatment to control) against −log10 p value (G, HG vs Control; H, HG+rVEGF vs Control; I, HG+ nitric oxide donor vs Control). The red dots represent the 143 peptide peaks that were dysregulated by hyperglycemia but returned to control levels by both rVEGF (H) and nitric oxide donor (I). The red horizontal lines are drawn at p = 0.05 and the red vertical lines are drawn at log_2_ ratio = 1 and −1. Scale bars = 100 µm. (A–B is reprinted from Am J Pathol 2001, 158:1199-206 with permission from the American Society for Investigative Pathology.)

Under normoglycemic conditions, control embryos exhibit normal organogenesis and vascular development of the yolk sac (Fig. 1A, whole embryo inside a functional yolk sac containing circulating red blood cells). Hyperglycemic embryos display malformation of the embryo proper and yolk sac vasculature (Fig. 1B, abnormal embryo with a nonfunctional vitelline circulation, evidenced by pooling of blood at one pole of the yolk sac). Whole mount staining of control yolk sacs for the endothelial marker PECAM-1 confirms the formation of a mature, remodeled vasculature with a hierarchy of arborizing large and small vessels (Fig. 1C) while hyperglycemic yolk sacs appear arrested at the primary capillary plexus stage of vascular development (Fig. 1D). By E9.5, endocardial cells (cardiac endothelial cells) of the endocardial cushions are actively undergoing EMT and invading the cardiac jelly in control embryos (Fig. 1E). Hyperglycemia results in an acellular endocardial cushion due to inhibition of EMT, which prematurely arrests cushion morphogenesis (Fig. 1F).

We hypothesized that the hyperglycemic insult may be used to enrich for factors involved in endothelial differentiation/dysfunction. Due to the global biological effects of hyperglycemia, we expected a large number of proteins to exhibit expression changes, but only a subset to be relevant to endothelial differentiation/dysfunction. Therefore two additional groups, “rescue conditions”, were included in the experimental design. Our previous studies demonstrated that exogenous rVEGF-A_165_ or a nitric oxide donor supplementation reverses the morphological, functional and biochemical changes induced by hyperglycemia in the cardiovascular system [23], [36], [37]. Thus, we hypothesized that the proteins dysregulated by hyperglycemia but returned to normal levels by both rVEGF and nitric oxide donor represent a set of proteins that are important for normal vascular development.

### Liquid Chromatography Mass Spectrometry (LC-MS) of Yolk Sac Tissue

Groups of ten 7.5 dpc conceptuses were cultured in normoglycemia, hyperglycemia, hyperglycemia plus rVEGF-A_165_, or hyperglycemia plus nitric oxide donor. Yolk sacs were harvested at 24 hours, at the end of the primary capillary plexus stage and beginning of the vascular remodeling stage, and processed individually for mass spectrometry. LC-MS detected 39,120 peaks which were subsequently analyzed using MZmine software [24], [25]. To increase confidence, a data reduction step discarded peaks detected in less than 5 samples resulting in 12,908 peptide peaks in the intensity matrix (Table S1). Data alignment, normalization and statistical treatment revealed 651 differentially regulated peaks between normal and hyperglycemic samples. Among those, 143 were dysregulated (upregulated or downregulated) by hyperglycemia and returned to control levels by both the rVEGF and nitric oxide donor groups. These 143 peaks were targeted (in an aliquot of the original yolk sac lysate sample) for MS-MS fragmentation and subsequent protein identification using the Mascot search engine (www.matrixscience.com) and NCBInr Database.

To globally visualize the dataset, volcano plots were generated depicting each of the 12,908 peptide peaks as a point and plotting the log_2_ ratio (of treatment group to control) against −log10 p value (Fig. 1G–I). The red dots in the volcano plots represent the 143 peptide peaks that are dysregulated by hyperglycemia [inclusion criteria: both p<0.05 and log_2_ (fold change)>1] but returned to control levels by both rVEGF and nitric oxide donor [inclusion criteria: p>0.05 or p<0.05 and log_2_ (fold change)<1]. Significant peptide peaks were ranked by fold change and those greater than or equal to 2 [log_2_ (fold change)≥1] were included. Of the 143 peptide peaks, approximately 75% were upregulated. The majority of peptide peaks display a 2–4 fold change [log_2_ (fold change)≥1–2]. Though these changes appear minor, in developmental contexts slight changes in growth factors or signaling pathways disrupt cardiovascular development. For instance, a one fold increase or half fold decrease in VEGF leads to lethal cardiac defects [38].

### Protein Identification of the Subset of Peptide Peaks Dysregulated by Hyperglycemia But Returned to Normal Levels by Both rVEGF and Nitric Oxide Donor

Candidate proteins with the highest Mowse score [39] were mapped onto Gene Ontology classes in order to group proteins according to biological processes (Table 1) [40]. Candidates included distinct collagen and laminin chains which are known to surround vessels and provide environmental cues to endothelial cells [41]. Extracellular matrix (ECM) and provisional matrices, including the cardiac jelly, contain laminin, collagen and other ECM molecules many of which are also known to be expressed in human amniotic fluid (AF). Interestingly, patients with diabetic retinopathy secrete into the circulation a detectable increase in the level of laminin, suggesting that it may be a useful biomarker [42]. Proteoglycans are also present in the vasculature and the endocardial cushions, therefore it was not surprising to find chondroitin 6 sulfotransferase 3 (CHST3), an enzyme that sulfates proteoglycans, as one of the dysregulated proteins in our dataset.

**Table 1:** Candidate Proteins Associated with the Subset of Statistically Significant Peptide Peaks Dysregulated by Hyperglycemia But Returned to Normal Levels by Both rVEGF and Nitric Oxide Donor.

Category	Protein Name	Entrez GeneID	Gene Ontology Processes
**Adhesion/ Migration**	Chemokine (C-X-C motif) ligand 15 (Cxcl15)	20309	Chemotaxis; hemopoiesis; signal transduction; immune response
	Chondroitin 6-sulfotransferase (Chst3)	53374	Carbohydrate metabolic process
	Collagen, type IV, alpha 2 (Col4a2)	12827	Cell adhesion; phosphate transport
	Collagen, type VII, alpha 1 (Col7a1)	12836	Cell adhesion; phosphate transport
	Laminin, alpha 4 chain (Lama4)	16775	Cell adhesion; blood vessel development; regulation of embryonic development
	Matrix metalloprotease 2 (Mmp2)	17390	Blood vessel maturation; collagen catabolic process; proteolysis
	NOGO-A (Rtn4)	68585	Regulation of cell migration; angiogenesis; nervous system development
	Proprotein convertase 1 (Pcsk1)	18548	Peptide biosynthetic process; proteolysis
	Suppressor of tumorigenicity 14 (St14)	19143	Cell migration; proteolysis
**Cytoskeleton**	Dishevelled associated activator of morphogenesis 2 (Daam2)	76441	Actin cytoskeleton organization and biogenesis
	Filamin C, gamma (actin binding protein 280) (Flnc)	68794	Actin filament-based process
	Kinesin family member 21B (Kif21b)	16565	Microtubule-based movement
	Tubulin, alpha 1B (Tuba1b)	22143	Microtubule-based movement
**Differentiation/ Development**	E74-like factor 5 (Elf5)	13711	Ectoderm development; transcription
	Hairy enhance of spilt 6 (Hes6)	55927	Cell differentiation; transcription
	Jumonji, AT rich interactive domain 2 (Jarid2)	16468	Multicellular organismal development; transcription
	Laminin, alpha 4 chain (Lama4)	16775	Blood vessel development; cell adhesion; regulation of embryonic development
	Notch1	18128	Cell differentiation; heart development; EMT; angiogenesis
	NOGO-A (Rtn4)	68585	Nervous system development; angiogenesis; regulation of cell migration
	Protein inhibitor of activated STAT, 4 (Pias4)	59004	Multicellular organismal development; JAK-STAT cascade; transcription; protein sumoylation
	Single-minded 2 (Sim2)	20465	Cell differentiation; nervous system development; transcription
	WNT16	93735	Multicellular organismal development
**Insulin Signaling**	Proprotein convertase 1 (Pcsk1)	18548	Peptide biosynthetic process; proteolysis
	Protein tyrosine phosphatase, non- receptor type 1 (Ptpn1)	19246	Insulin receptor signaling pathway; Dephosphorylation
	Zinc finger protein 106 (Zfp106)	20402	Insulin receptor signaling pathway
	Adaptor-related protein complex 3, beta 1 subunit (Ap3b1)	11774	Antigen processing/presentation; protein transport; endocytosis
	Chemokine (C-X-C motif) ligand 15 (Cxcl15)	20309	Immune response; chemotaxis; hemopoiesis; signal transduction
	Interleukin 25 (Il25)	140806	Inflammatory response
**Proteolysis**	A disintegrin and metalloproteases 15 (Adam15)	11490	Proteolysis; integrin-mediated signaling
	Proprotein convertase 1 (Pcsk1)	18548	Proteolysis; peptide biosynthetic process
	Matrix metalloprotease 2 (Mmp2)	17390	Proteolysis; blood vessel maturation; collagen catabolic process
	Suppressor of tumorigenicity 14 (St14)	19143	Proteolysis; cell migration
**Signal Transduction**	A disintegrin and metalloproteases 15 (Adam15)	11490	Integrin-mediated signaling; proteolysis
	Chemokine (C-X-C motif) ligand 15 (Cxcl15)	20309	Signal transduction; chemotaxis; hemopoiesis; immune response
	Protein inhibitor of activated STAT, 4 (Pias4)	59004	JAK-STAT cascade; multicellular organismal development; transcription; protein sumoylation
	Protein kinase C binding protein 1 (Prkcbp1)	228880	Phosphorylation
	Protein tyrosine phosphatase, non- receptor type 1 (Ptpn1)	19246	Dephosphorylation; insulin receptor signaling pathway
**Transcription/ Translation**	DEAD (Asp-Glu-Ala-Asp) box polypeptide 31 (Ddx31)	227674	RNA processing
	E74-like factor 5 (Elf5)	13711	Transcription; Ectoderm development
	Hairy enhance of spilt 6 (Hes6)	55927	Transcription; Cell differentiation
	Jumonji, AT rich interactive domain 2 (Jarid2)	16468	Transcription; Multicellular organismal development
	Protein inhibitor of activated STAT, 4 (Pias4)	59004	Transcription; JAK-STAT cascade; multicellular organismal development; protein sumoylation
	Ribosomal protein L18 (Rpl18)	19899	Translation
	Serine/arginine repetitive matrix 2 (Srrm2)	75956	RNA splicing
	Single-minded 2 (Sim2)	20465	Transcription; cell differentiation; nervous system development
	SRY-box containing gene 19 (Sox19)	20673	Regulation of transcription
**Transport**	Adaptor-related protein complex 3, beta 1 subunit (Ap3b1)	11774	Protein transport; endocytosis; antigen processing/presentation
	ATPase type 13A1 (Atp13a1)	170759	Cation transport; metabolic process
	ATP-binding cassette transporter, sub- family A, member 13 (Abca13)	268379	Transport; protein targeting
	ATP-binding cassette, sub-family A, member 7 (Abca7)	27403	Transport; phagocytosis
	Collagen, type IV, alpha 2 (Col4a2)	12827	Phosphate transport; cell adhesion
	Collagen, type VII, alpha 1 (Col7a1)	12836	Phosphate transport; cell adhesion
	Down syndrome critical region gene 3 (Dscr3)	13185	Vacuolar transport
	Solute carrier family 23 (nucleobase transporters), member 1 (Slc23a1)	20522	Ion transport
	Transcobalamin 2 (Tcn2)	21452	Ion transport
	Transient receptor potential protein 5 (Trpc5)	22067	Ion transport
**Others/Unknown**	Enolase 1, alpha non-neuron (Eno1)	13806	Glycolysis
	F-box protein 42 (Fbxo42)	213499	Ubiquitin cycle
	TNF receptor-associated protein 1 (Trap1)	68015	Protein folding; response to stress
	Microrchidia 2a (Morca2a)	74522	?
	T complex expressed gene 4 (Cramp1l)	57354	?
	T complex protein-10 (Tcp10)	21458	?
	Tetratricopeptide repeat domain 15 (Ttc15)	217449	?
	Tripartite motif containing 68 (Trim68)	101700	?

Several proteins in our dataset mapped to the Gene Ontology term “Proteolysis”. These proteins function in varied biological processes involving proteolytic activity such as cell-cell adhesion, migration/invasion, matrix remodeling, growth factor release/activation, receptor shedding and cytokine signaling. The candidate proteases detected, included ADAM15, MMP2, Suppressor of tumorigenicity 14 (ST14) and Proprotein convertase 1 (Pcsk1).

A number of candidate proteins that directly affect differentiation and embryonic development were discovered in our dataset, including Hairy and enhancer of split 6 (HES6), Notch1, NOGO-A, Jumonji, and WNT16. Several other candidate proteins identified in our screen mapped to the Gene Ontology term “Transport” including Transient receptor potential protein (TRPC5), a calcium channel, Sodium/vitamin C transporter (SVCT or Slc23a1), a sodium/ascorbate cotransporter, and ATP binding proteins (Atp13a1, Acca13, and Abca7). Other proteins of note found in our database include Fbxo42 (part of the ubiquitin ligase complex), Protein inhibitor of activated STAT4 (PIASγ) (a SUMO ligase), Kinesin-like protein KIF21b (a microtubule binding motor protein), Enolase 1 (an enzyme in the glycolysis pathway) and Microrchidia 2a (Morc2a or ZCWCC1).

Several candidate factors in our dataset have been correlated with disease states including the Ets family member Elf5, which is upregulated in breast cancer [43]. Interestingly, the bHLH transcriptional repressor Single-minded 2 which is thought to contribute to the etiology of Down syndrome [44] and Down syndrome critical region gene 3 (DSCR3), located on human chromosome 21, were present in our dataset. Approximately, 40–50% of infants with Down syndrome have CHDs. Finally, protein tyrosine phosphatase, non-receptor type 1 (PTPN1) was identified in our dataset. PTPN1 plays a role in obesity and metabolism. Further, gene variants have been identified in obese people and type 2 diabetics [45], [46], [47].

**Figure 2:**
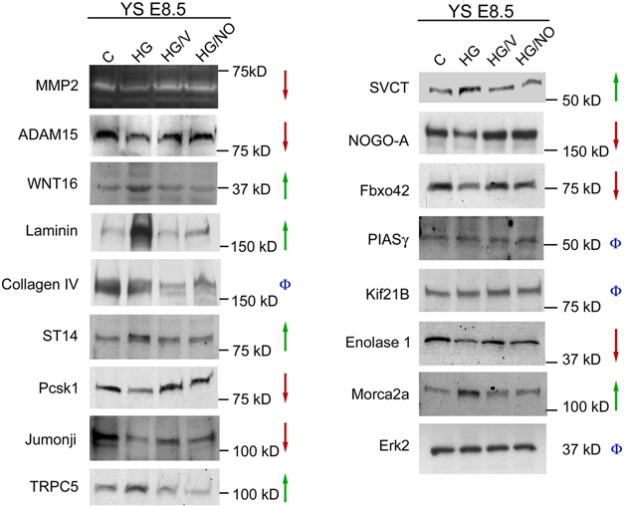
Validation of Differential Expression of Candidate Proteins Identified in the Proteomic Profiling Study. To validate candidate proteins detected by LC-MS-MS, Western blotting and zymography was performed on lysates of yolk sacs harvested from embryos cultured under the four experimental conditions. Erk2 was used as a loading control. Green arrows represent protein levels that increased with hyperglycemia (HG) treatment but returned to control levels in both rescue conditions (HG/V and HG/NO). Red arrows represent protein levels that decreased with hyperglycemia treatment but returned to control levels in both rescue conditions (HG/V and HG/NO). The Phi symbols represent protein levels that did not vary across treatment groups or did not vary in the expected pattern (increased or decreased by HG and returned to control levels in both rescue conditions).

In order to assess the biological significance of the dysregulation of these candidate proteins and validate our MS-MS results (Fig. 2), Western blotting was performed on lysates of yolk sacs harvested from embryos cultured under the four experimental conditions. ADAM15, Pcsk1, and NOGO-A were confirmed to be downregulated while Laminin, TRPC5, WNT16, Morc2a, and CHST3 were confirmed to be upregulated. Additionally, a reduction in the proteolytic activity of MMP2 was observed by zymography. After hyperglycemia treatment, the change in expression of most proteins was 2 fold: densitometry of the blots displayed in Figure 2 detected a fold change decrease of 1.9 for MMP2, decrease of 2.7 for ADAM15, increase of 2 for ST14, and decrease of 1.9 for Pcsk1 in the HG group. These differences are consistent with the mass spectrometry analysis (Figure 1G–I and Table S1) which revealed that the majority of peptide peaks display a 2–4 fold change. Additional proteins that displayed the expected fold change included WNT16, Jumonji, TRPC5, SVCT, NOGO-A, Fbxo42, Enolase1, and Morca2a. The only protein that validated but did not demonstrate the predicted fold change was Laminin. These data contribute to the accumulating evidence of the accuracy of label-free LC-MS measurements to predict relative protein changes [48], [49]. Several identified proteins did not validate by Western blot and were not pursued further. Several other proteins were not pursued because no commercially available antibodies were available for validation and subsequent investigation.

We discovered a number of unnamed and predicted proteins with significant dysregulation. Continued curation of the public databases will reveal the identities and functions of unnamed proteins identified from cDNA initiatives; however, as little information is currently available on such proteins these were not further pursued. Many proteins were identified from multiple different significant peptide peaks (i.e. different fragments within the same protein). The consolidated list consisting of 44 proteins is presented in Table 1 (excluding unnamed and predicted proteins).

### Factors Involved in Yolk Sac Vascular Development Are Also Important for Cardiac Development

To validate our assumption that proteins relevant to cardiac development may be isolated from vascular tissue, proteins from the validated candidate list were tested by immunofluorescence microscopy for their presence in the endocardial cushion at E9.5 (a stage of cardiac development when EMT is actively occurring). This is a time and place during cardiogenesis that is widely recognized to be a “hot spot” of cardiac anomalies.

**Figure 3:**
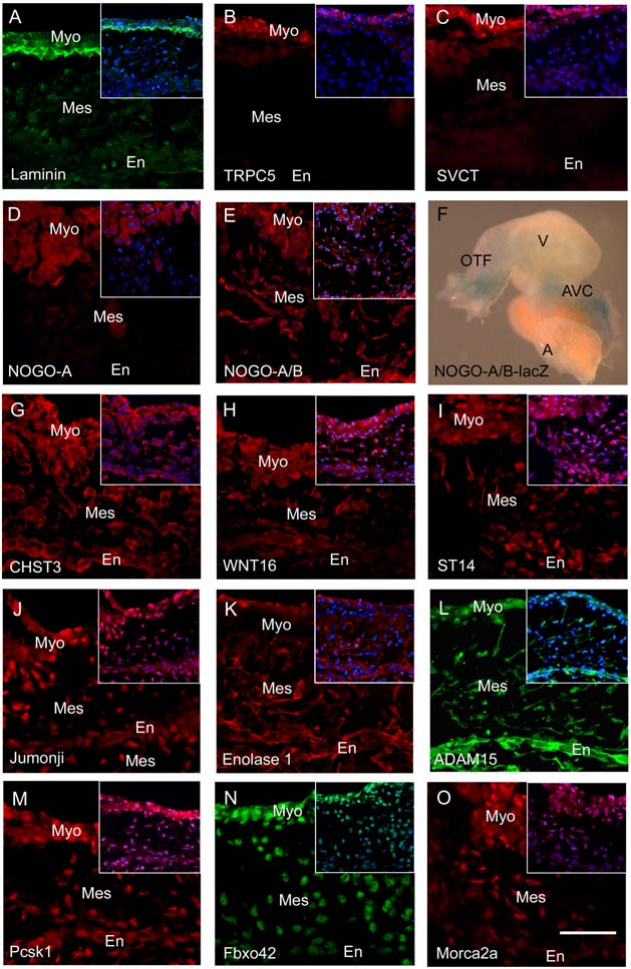
Spatial Expression Pattern of Validated Proteins in *in vivo* Embryonic Hearts. Immunofluorescence (red or green) was performed on hearts excised at E9.5 to confirm that the validated proteins identified in the vascular dataset are also present in the heart (A–E, G–O). Insert contains the DAPI (blue) merged image. F: Whole mount β-gal staining of an E9.5 NOGO-A/B-lacZ heart. Outflow Tract (OFT); Ventricle (V); AVC (Atrioventricular Canal); A (Atrium); Myocardium (Myo); Endocardium (En); Mesenchyme (Mes); Scale bar = 50 µm.

Laminin 1 was strongly present at the interface of the myocardial and mesenchymal zones of the cushion, consistent with basement membrane localization (Fig. 3A). TRPC5 and SVCT were highly expressed in the compact myocardium and weakly/absent throughout the rest of the myocardium and cushion (Fig. 3B, C). Potentially these factors play a role in contraction or function of the valves. NOGO-A localizes strongly within the myocardium while staining with a NOGO-A/B antibody reveals localization within the mesenchymal and endothelial cells, suggesting NOGO-B presence in these cell types (Fig. 3D, E). β-galactosidase staining of transgenic lacZ NOGO-A/B whole hearts harvested at E9.5 demonstrated localization to the outflow tract and atrioventricular canal (Fig. 3F). CHST3, WNT16, and ST14 were detected throughout the cell layers of the endocardial cushion and in the myocardium (Fig. 3G–I). Jumonji was present strongest in the myocardium and mesenchyme, and weakly in the endocardium (Fig. 3J). The expression pattern of Enolase 1 was localized to the endocardium, mesenchyme, and epicardium, but absent from the myocardium (Fig. 3K). ADAM15 was highly expressed in the endocardium and mesenchymal cells, but also present in the myocardium (Fig. 3K, L). NOGO-B, ST14, Enolase 1, and ADAM15 had a notable presence within the cellular projections of the mesenchymal cells. Immunoreactivity for Pcsk1 was strongest in the myocardium and weaker in the endocardium and mesenchyme (Fig. 3M). Fbxo42 displayed nuclear expression in a gradient pattern across the layers of the cushion to the myocardium (Fig. 3N). The zinc finger protein, Morc2a, was present in an increasing gradient from the endocardium to the mesenchymal cells to the myocardium, and most intensely in a nuclear/perinuclear pattern (Fig. 3O).

**Figure 4:**
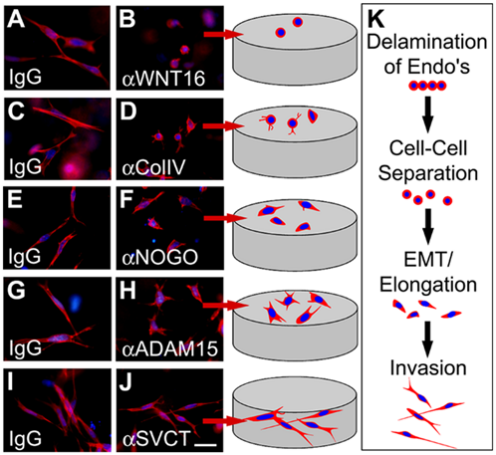
Analysis of the Potential Biological Function of Identified Proteins in an *ex vivo* Cardiac Assay. The potential biological effects of identified proteins were characterized in the *ex vivo* atrioventricular canal assay to determine if proteins relevant to cardiac development may be isolated from the vascular dataset. Control IgG (A, C, E, G and I) or antibodies directed against WNT16 (B), Collagen IV (D), NOGO-A/B (F), ADAM15 (H), and SVCT (J) were added to the media bathing the explants. After 48 hours F-Actin (red) staining was performed and a high magnification image of a few representative cells was taken (blue = DAPI). Cartoons representing the various stages of EMT (K) and the morphology of the cells within the high magnification image from each treatment are displayed. Scale bar = 50 µm.

Proteins were selected for further study in a functional assay, the *ex vivo* atrioventricular canal assay, that has yielded many insights into the molecular events that underlie endocardial cushion development [4]. The atrioventricular canal assay mimics the spatiotemporal, molecular and biochemical events that occur during *in vivo* valve development and provides a system amenable to experimental manipulation [50], [51]. In this model, the atrioventricular canal segment is excised prior to EMT; the cushion tissue is exposed and placed onto the surface of a collagen gel. Media containing preservative free antibodies directed against secreted proteins or the extracellular domain of membrane proteins is added to the cultures. The endothelial cells migrate onto the surface of the gel and away from the explants. In response to inductive signals secreted by the myocardium a subset of the endothelial cells undergo EMT and invade into the gel. Cells undergoing EMT follow a semi-linear progression of morphological changes including: 1) lateral cell-cell separation from epithelial sheets, 2) elongation of the single, rounded cells on the surface of the gel, and 3) invasion of elongated cells into the gel (Fig. 4K). Approximately 300 cells migrate out of each of the explants, however in order to observe the cellular morphology in detail, a high magnification image of few cells is displayed in Figure 4. Additionally, a cartoon representation of these cells is provided to highlight the varied and detailed cellular morphologies observed in this assay.

Normal cultures (IgG treatment) exhibited the characteristic phenotype of EMT, displaying elongated, invasive mesenchymal cells (Fig. 4A, C, E, G and I; images taken below the gel surface). However a dramatic inhibition of cardiac EMT resulting in the majority of cells maintaining an epithelial phenotype (rounded and non-invasive) was observed with WNT16 sequestration (Fig. 4B; gel surface image). In the yolk sac, an up regulation of WNT16 was associated with mal-development (abnormal increase in endothelial cells) while in the atrioventricular canal an inhibition of WNT16 was associated with decreased differentiation, suggesting WNT16 is a positive regulator of differentiation. Inhibition of cell adhesion using a collagen IV antibody led to the formation of epithelial cells with a rounded morphology and the presence of short cellular projections (Fig. 4D; gel surface image).

Blocking NOGO binding using a NOGO-A/B antibody directed against its extracellular domain revealed a novel role for NOGO in cardiac development. The treated cells appear to undergo a partial EMT or prematurely arrested EMT as evidenced by the formation of oval cells rather than fully elongated cells (Fig. 4F; gel surface image). However, given the known roles of NOGO in the neuronal system, a guidance or matrix interaction defect may underlie the observed phenotype. At this time it is unclear if this effect is due to NOGO-A or NOGO-B as the antibody used in these studies detects both isoforms. NOGO-A was the isoform identified in our MS dataset however our localization data suggest NOGO-B is expressed by the endocardium and mesenchyme. Further, a recent paper demonstrated a role for NOGO-B in vascular cell biology [52].

Atrioventricular canals cultured in the presence of an antibody directed against the ectodomain of ADAM15 resulted in blunted cardiac EMT (Fig. 4H; gel surface image) compared to IgG control. The cells begun to elongate and displayed multiple cellular extensions in a star-like pattern, but failed to complete elongation and invade the gel. Antibody blocking of the extracellular domain of SVCT failed to reveal a role for this transporter in EMT processes (Fig. 4J; image taken below the gel surface). Collectively, these results demonstrate that several proteins in our dataset are present in the embryonic heart and have novel roles at various steps of cardiac EMT, confirming that our approach utilizing vascular tissue did identify proteins relevant to cardiac development.

### Identification of Biomarkers of Human Congenital Heart Defects

One of the aims of this study was to identify biomarkers that discriminate diseased from healthy fetuses using the proteomic dataset as a guide for candidate proteins that require follow up with more precise protein chemistry methods (Western blot, immunoflourescence microscopy, and explant assays). A pilot study was initiated to determine if the expression of these proteins vary in the AF of a cohort of healthy, non-diabetic pregnant women. AF was obtained from routine amniocentesis of women carrying normal fetuses (n = 17) and women carrying fetuses with CHDs (n = 16) (population details in Materials and Methods). The levels of candidate biomarkers in the AF were assessed by Western blot followed by densitometry.

**Figure 5:**
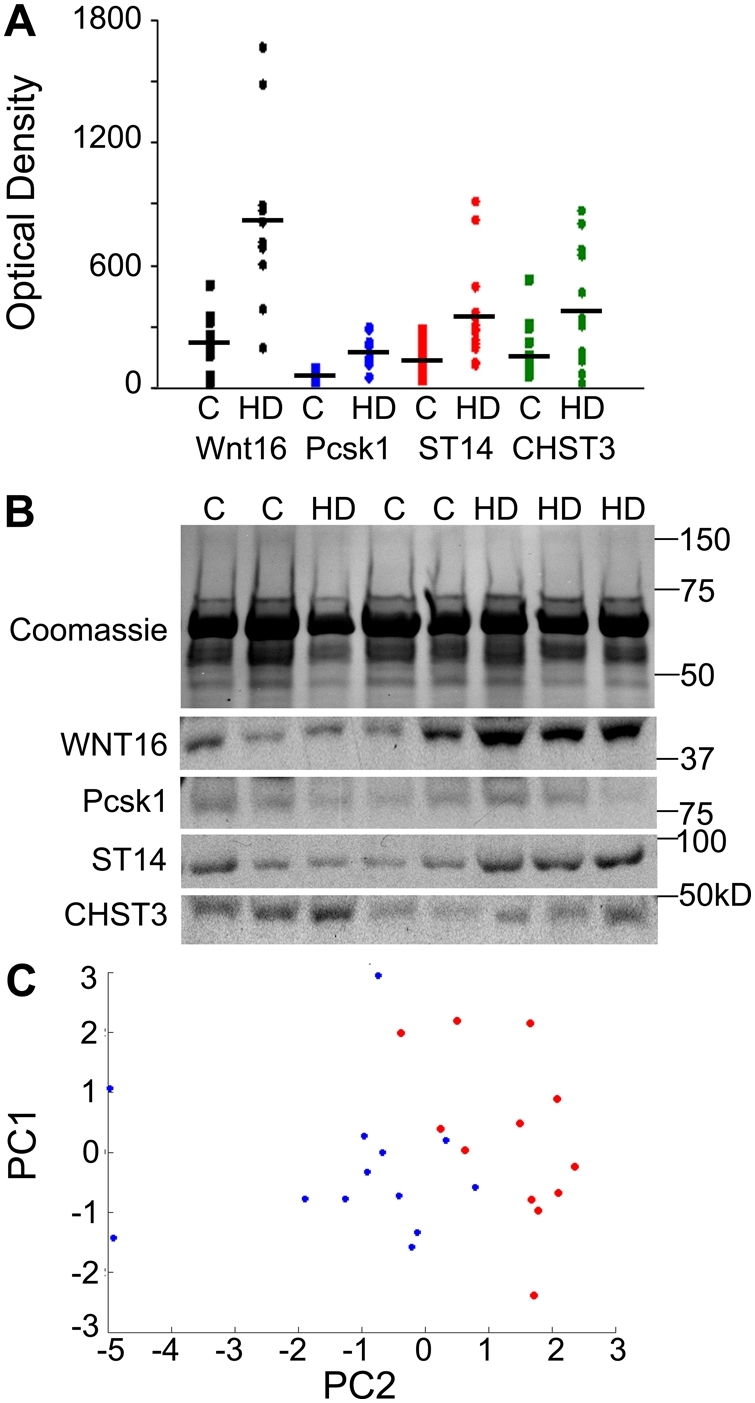
Identification of Biomarkers of Human Congenital Heart Defects. Densitometry data (A) from Western blots of AF [CHDs (HD) N = 17; Controls (C) N = 16]; black bars represent the mean. Coomassie stained gel of AF samples and representative Western blots for WNT16, Pcsk1, ST14, and CHST3 on a sampling of patients (B). PCA plot (C) for all biomarkers demonstrates separation of CHDs samples from controls. Red (Control); Blue (CHD).

CHST3 was significantly (p<0.01) increased in the AF of women carrying fetuses with CHDs (mean = 380) compared to controls (mean = 176) by Student's t-test (unpaired; two-tailed distribution; two-sample equal variance), but not significant by univariate logistic regression (p<0.1; misclassification error of 0.33) (Fig. 5A, B; Table 2). Morca2a demonstrated an increased trend in the AF of women carrying fetuses with CHDs (mean = 505) compare to controls (mean = 374); however, this finding was not significant by t-test (p<0.1) nor univariate logistic regression (p<0.2, misclassification error of 0.35).

**Table 2:** Univariate Logistic Regression and Misclassification Error for Amniotic Fluid Biomarkers.

Marker	Coefficient	*p*-Value	OR	95% CI	MC error
Wnt16	2.71	0.006	15.01	2.16–104.44	0.11
SVCT	0.62	0.252	1.86	0.64–5.35	0.41
Pcsk1	3.29	0.012	26.95	2.07–350.70	0.12
CHST3	0.51	0.129	1.67	0.86–3.22	0.33
Enolase	−0.43	0.213	0.65	0.33–1.28	0.59
PIAS	0.46	0.209	1.58	0.77–3.21	0.63
ST14	1.97	0.013	7.17	1.51–34.14	0.19
Morca2a	0.58	0.213	1.78	0.72–4.41	0.35

The biomarkers that showed significant association with CHDs in univariate logistic regression were WNT 16, Pcsk1 and ST14 (Table 2). WNT16 was significantly increased (p = 0.006) in the AF of women carrying fetuses with CHDs (mean = 818) compared to controls (mean = 220) as was Pcsk1 (p = 0.012; mean = 167 compared to control mean = 50), and ST14 (p = 0.013; mean = 356 compared to controls mean = 135) (Fig. 5A, B). In a cross-validation experiment, these markers were able to correctly classify controls from cases in up to 89% of cases (misclassification error of 0.11). Proteins that did not appear to be predictive for heart defect included SVCT, Enolase and PIASγ. PCA analysis using all biomarkers revealed clustering of controls and cases (Fig. 5C) demonstrating that the CHD samples segregate from the controls. Although a larger cohort is required to confirm the specificity and sensitivity of these markers for diagnosis, our pilot study of human AF revealed promising biomarkers able to detect a range of CHDs (ventricular septal defects, valvular stenosis, complete atrioventricular septal defects, tetralogy of Fallot, and transposition of the great arteries) that in part result from defects in cushion morphogenesis. Furthermore, dysregulation of WNT16, ST14 and Pcsk1 may reflect the molecular origins of CHDs.

## Discussion

Applying a proteomic approach to embryonic vascular tissue generated from an *ex vivo* model of vasculopathy, we have identified proteins and pathways that are disrupted during cardiovascular development and lead to the observed pathological phenotype. Dysregulation of this set of proteins in a “normal” fetus may signal that the fetus is congenitally at risk or encountering an environmental stressor. Several of these protein targets (WNT16, Pcsk1 and ST14) provided the basis for a novel prenatal screen for the detection of CHDs, suggesting dysregulation of WNT16, ST14 and Pcsk1 may play a role in the etiology of human CHDs. Several forms of CHDs are known to have a genetic basis such as microdeletions (22q11) in DiGeorge Syndrome and single gene mutations (*JAG1*) in Alagille Syndrome. It would be of interest to determine if individuals with CHDs carry genetic mutations in *WNT16*, *ST14* or *Pcsk1*. At the protein level, these markers are overexpressed therefore if sequence variants are found mutations would likely affect protein function and/or activity. In the case of ST14 and Pcsk1, both of which are enzymes, a hypomorphic or hypermorphic mutation in these genes has the potential to affect multiple substrates and hence several cellular processes.

**Figure 6:**
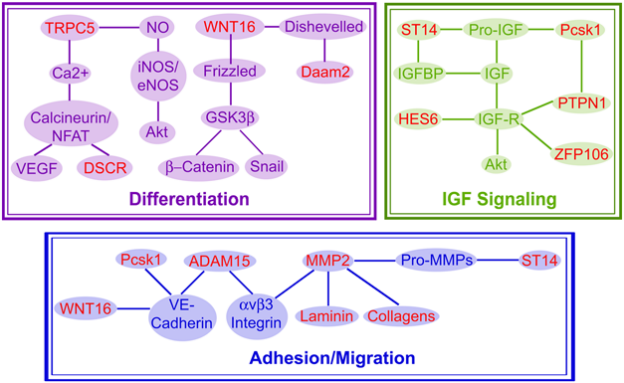
Integration of Proteins Identified by Proteomic Profiling with Known Pathways and Biological Processes Involved in Cardiovascular Development. We identified proteins (in red) known and previously unknown to have roles in cardiovascular development with diverse functions including migration, differentiation, IGF signaling, protease activity and transport (Table 1). Several of the proteins interact upstream or downstream of each other and with established factors in cardiovascular development. Dysregulation of these proteins/pathways not only reflect the molecular origins of CHDs but, in a normal fetus, may signal that the fetus is at risk or encountering an environmental stressor thus providing the basis for novel diagnostic tests.

Changes in cellular processes not only reflect the molecular origins of disease but also provide avenues for molecular therapies to ameliorate the defect. To this end, we identified several proteins, previously unknown to have a role in cardiovascular development, with diverse functions that cluster to adhesion/migration, differentiation, transport, IGF signaling pathways (Table 1). Although we discovered and characterized several proteins with currently unknown roles in cardiovascular development, we were able to place them in their relevant biological context by relating them to existing functional cascades (signaling, enzymatic, polarity, migration, and differentiation). Protein networking analysis revealed that several of the identified proteins interact upstream or downstream of each other and with established factors in cardiovascular development (Fig. 6). The presence of proteins known to have roles in cardiac development within our dataset further validates our approach. For example, our screen detected the transcriptional repressor Jumonji whose deletion is known to result in myocardial defects and ventricular septal defects [53]. However our data demonstrating decreased Jumonji during vasculopathy extends the role of Jumonji to vascular development.

Previously, we demonstrated that yolk sac vasculopathy is associated with increased nitric oxide, however the nitric oxide targets remained elusive [36]. Recently nitrosylation sites on the cytoplasmic domain of TRPC5 were identified and in response to nitric oxide, Ca(2+) entered bovine aortic endothelial cells [54]. In this study we observed increased TRPC5, raising the possibility that the observed yolk sac vasculopathy is mediated by nitric oxide driven over-activation of TRPC5. We speculate that the subsequent abnormal influx of calcium through TRPC5 activates the calcineurin/NFAT pathway [55]. In the developing heart, the NFAT pathway is tightly regulated and has complex interactions with DSCR (another protein identified in our screen) and VEGF to drive differentiation; low levels of VEGF are required for EMT while high levels terminate it [56], [57]. Therefore, it is tempting to speculate that TRPC5 is a novel player in cardiac development, acting upstream of NFAT, leading to dysregulation of VEGF expression and ultimately termination of heart development.

On the other hand, positive regulation of cardiac EMT relies on Wnt/β-catenin signaling however the specific Wnts involved are largely unknown with the exception of Wnt9a in the avian heart [58]. Variations in Wnt signaling are expected to have direct effects on valve plasticity as canonical Wnt/β-catenin signaling induces mesenchymal differentiation and modulates the amount of mesenchyme produced [59], [60]. One defect associated with vasculopathy is increased endothelial cell number per vessel, leading to a hypertrophic, nonfunctional capillary plexus. Thus the observed upregulation of WNT16 may lead to unregulated endothelial cell differentiation or proliferation, ultimately affecting the organization of vascular structures. Concomitantly, antibody neutralization of WNT16 blunts cardiac EMT in the atrioventricular canal assay. Though the WNT16 isoform is known to be expressed in the embryonic heart [61], a functional role for WNT16 has not been previously reported, thus our atrioventricular canal assay results describes a new function for a novel WNT member in the cardiac developmental cascade. Additionally, we observed increased WNT16 in human amniotic fluid (AF) of women carrying fetuses with CHDs and demonstrated that this is predictive of CHDs. Monitoring WNT16 levels and targeting WNT16 signaling may provide novel avenues for future diagnostic and pharmacological research.

Proteoglycans and extracellular matrix are important players in cell adhesion, migration, proliferation, and angiogenesis, processes important in both development and disease. CHST3 catalyzes the transfer of sulfate to position 6 of the N-acetylgalactosamine (GalNAc) residue of chondroitin. Human endothelial cells express CHST3 mRNA while endothelial cells overlying the porcine mitral valve produce chondroitin sulphate [62], [63]. Interestingly, gain of function experiments with the heart development regulatory gene *Tbx20* in the avian endocardial cushion resulted in decreased chondroitin sulfate proteoglycans and increased mesenchyme [64]. Our data demonstrating CHST3 expression in the murine endocardial cushion suggests that this may be a conserved mechanism to regulate mesenchyme production, although further studies are required to confirm this hypothesis. Though not predictive of CHDs, CHST3 was dysregulated in the AF of woman carrying fetuses with CHDs, suggesting a role in cardiac development.

An additional factor involved in adhesion/migration identified in our study is the protease ADAM15. Multiple individual ADAM isoform KO mice display cardiac defects, including Adam 9, 17, and 19 [65], [66], [67]. ADAM15 is expressed in blood vessels and endocardium, and although ADAM15 mice are viable studies using retinopathy and tumor angiogenesis models demonstrate an inducible vascular phenotype (failure of remodeling of the primary capillary plexus) [68]. In conjunction, our data indicates that decreased ADAM15 enzymatic activity in the hyperglycemic yolk sacs disrupts growth factor/cytokine signaling of a yet unknown pathway, resulting in an inability of the endothelial cells to initiate remodeling, consistent with the previous report on an inducible vascular phenotype. Our data from the atrioventricular canal assay extends the known functions of ADAM15 to include cardiac EMT. Speculating on the substrates involved in its effects on differentiation to mesenchymal cells, ADAM15 proteolytic activity on cadherins is one plausible target [69]. Further, the strong immunofluorescent staining of ADAM15 in the cellular projections of cardiac mesenchymal cells suggests ADAM15 may be necessary for cell interaction with the matrix potentially via integrin binding. These findings warrant future studies to determine if ADAM15 is predicative of CHDs. Interestingly, ADAM12 is a known biomarker for Down syndrome, demonstrating the applicability of this family to biochemical assays for diagnosis [70]. The novel finding of a functional role for ADAM15 in the embryonic heart combined with its strong expression in the cardiovascular system make it an appealing target for future studies that isolate its downstream targets in the heart.

In addition to proteins involved in differentiation and adhesion/migration, several proteins involved in the IGF/insulin pathway were identified in our study. Early in embryogenesis the embryo does not produce insulin nor does maternal insulin cross the placental barrier. However, embryos express high levels of IGF receptors and early growth is mediated via IGF signaling through Akt. Factors that regulate IGF receptor engagement and its downstream signaling cascades were identified both on the extracellular face, including the proteases Pcsk1 and ST14, and the intracellular face, including ZFP106 (or son of insulin receptor mutant) and HES6. Pcsk1 is a secreted protease that cleaves precursors including neuropeptides, renin, somatostatin, and insulin/IGF [71]. Additionally substrates include adhesion molecules, proteases and receptors. Pcsk1 KO mice are viable, but growth retarded. Two strains of proprotein convertase KO mice, Furin and PACE4, exhibit defects in the vasculature and heart, while the other 8 proprotein convertase KO mice exhibit defects in hormone production, fertility, and lipid metabolism [71]. Our data demonstrating decreased Pcsk1 levels in the yolk sac after hyperglycemia insult suggests that Pcsk1 KO mice may have an unappreciated inducible vascular phenotype. We also demonstrated that Pcsk1 is predictive for CHDs, expressed in the embryonic myocardium, and increased in the AF of fetuses with CHDs. Pcsk1 downregulation in the yolk sac and upregulation in AF may reflect differences in developmental age, cell differentiation processes or clearance/accumulation of proteins in the AF versus tissue. Although proprotein convertases have been linked to Alzheimer's disease, tumorigenesis, and infections, they have not been previously reported to have a role in CHDs. Further given that gene knock out experiments have determined that individual PCs do not seem to be redundant in embryonic development, understanding the role of Pcsk1 in metabolism, growth, and development of cardiovascular system is an intriguing avenue of future investigation.

Another molecule in the IGF signaling pathway, ST14, a transmembrane serine protease, has been implicated in metastatic cancer, which utilizes a molecular program reminiscent of embryonic EMT [72]. Substrates for ST14 include hepatocyte growth factor, urokinase-type plasminogen activator, protease-activated receptor 2 (PAR-2), IGF binding protein-related protein-1, trask, laminin and fibronectin [73], [74]. Recently, VEGFR-2 was reported to be a putative substrate of ST14 in HUVECs, resulting in ectodomain cleavage and inactivation of cell signaling [75]. Here, we demonstrated for the first time that ST14 is present in the embryonic cardiovascular system and is predictive of CHDs. Given the known substrates of this protease, ST14 induction during vasculopathy and in AF of fetuses with CHDs may affect differentiation, adhesion/migration and growth.

Cell movements, growth and differentiation depend on environmental homeostasis; of particular importance during development is redox balance. According to current knowledge, SVCT transports ascorbate into cells and thereby participates in metabolic processes, matrix synthesis and antioxidant defense [76]. Interestingly, dehydroascorbate utilizes glucose transporters to enter the cell prior to conversion to ascorbate, and during a hyperglycemic episode glucose competitively inhibits the transport of dehydroascorbate, potentially compromising antioxidant defense and leading to reactive oxygen species induced damage [77], [78]. Consistent with the theory of ROS induced vasculopathy; our data demonstrating upregulation of SVCT may signify that the embryo is “at risk” and employing a defense to combat oxidative stress. Although SVCT was not predictive of CHDs, the fetal cardiovascular system is sensitive to oxidative stress and benefits from antioxidant supplements, such as folic acid [13], [79], [80]. Therefore understanding the upstream regulation, downstream targets, and/or activity of SVCT may shed light onto novel antioxidant therapies for fetuses “at risk”.

Molecular-based diagnostic tests that detect fetuses “at risk” provide clinicians with information to guide clinical interventions aimed at improving outcome. First-trimester biochemical screening is becoming a powerful tool, allowing clinicians to efficiently triage patients [81]. For example, increased pregnancy-associated plasma protein A (PAPP-A) and decreased free β-human chorionic gonatrophin (β-hCG) in maternal serum indicate increased risk of trisomy 21, while reduced PAPP-A in maternal serum is associated with an increased risk of trisomy 18 [81]. An obvious advantage of serum screening is that it is non-invasive and serum screening may be performed earlier than amniocentesis. In the present study, we utilized AF rather than maternal serum because of its low complexity and similarity to the fetus due to its origin. Cells in AF are fetal rather than maternal. Future studies involving early maternal serum samples are required to determine the merit of adding our biomarkers to existing screening panels. To this end, using an *in vivo* streptozocin-induced diabetic model in mice, we detected differences in specific Laminin chains, ADAM15 ectodomain, and MMP2 activity in maternal sera from mice carrying a high proportion of fetuses with heart defects compared to those with a high proportion of fetuses with normal hearts (unpublished data). These data suggest that protein fragments are released into the maternal circulation in sufficient quantities that allow detection early in pregnancy (E10.5 which is approximately equivalent to human developmental week 6). Experiments are currently underway to evaluate the levels of WNT16, ST14, and Pcsk1 in maternal sera from murine models and humans.

The creation of new biochemical diagnostic tools to assay proteins such as Pcsk1, ST14, and WNT16 may give insights into the well-being of the fetus by sensing an inability to combat an environmental insult, disruption in growth, or damage due to reactive oxygen species. Affordable, rapid, and accurate molecular-based diagnosis of potential problems in fetal cardiovascular development early in pregnancy will provide clinicians with information to guide clinical interventions and pave the way toward eradicating one of the major global causes of infant mortality in the world. In the future, dietary supplementation or molecular therapies based on Pcsk1, ST14 and WNT16 or their targets may ameliorate the deficiencies that ultimately lead to the formation of a CHD in the first trimester. Therefore isolating the specific substrate targets of ST14 and Pcsk1 that disrupt cardiac development and investigating the transcriptional or post-transcriptional regulatory mechanisms that lead to overexpression of ST14 and Pcsk1 will aid in deciphering the role of these proteins in the etiology of CHDs. Further, continued mining of the proteomic dataset coupled with analysis of novel factors in physiologically relevant models will continue to yield fruitful avenues for investigation into the biology of cardiovascular development and pathogenic mechanisms of CHDs in the hope of advancing prevention, diagnosis and treatment.

## Supporting Information

Table S1 (2.75 MB XLS)
